# Effect of Dy substitution in the giant magnetocaloric properties of HoB_2_

**DOI:** 10.1080/14686996.2020.1856629

**Published:** 2021-01-22

**Authors:** Pedro Baptista de Castro, Kensei Terashima, Takafumi D. Yamamoto, Suguru Iwasaki, Ryo Matsumoto, Shintaro Adachi, Yoshito Saito, Hiroyuki Takeya, Yoshihiko Takano

**Affiliations:** a National Institute for Materials Science, Tsukuba, Japan; b University of Tsukuba, Tsukuba, Japan; c Hokkaido University, Sapporo, Japan

**Keywords:** Magnetic refrigeration, magnetocaloric effect, adiabatic temperature change, 203 Magnetics / Spintronics / Superconductors, Magnetocaloric Materials

## Abstract

Recently, a massive magnetocaloric effect near the liquefaction temperature of hydrogen has been reported in the ferromagnetic material HoB_2_. Here we investigate the effects of Dy substitution in the magnetocaloric properties of Ho_1-*x*_Dy*_x_*B_2_ alloys (*x* = 0, 0.3, 0.5, 0.7, 1.0). We find that the Curie temperature (*T*_C_) gradually increases upon Dy substitution, while the magnitude of the magnetic entropy change |Δ*S*_M_| and adiabatic temperature change Δ*T*_ad_ showed a gradual decrease. On the other hand, due to the presence of successive transitions in these alloys, the peak height of the above magnetocaloric properties tends to be kept in a wide temperature range, leading to a relatively robust figure of merit in a wide temperature span. These alloys could be interesting candidates for magnetic refrigeration in the temperature range of 10–60 K.

## Graphical abstract

**Figure:**
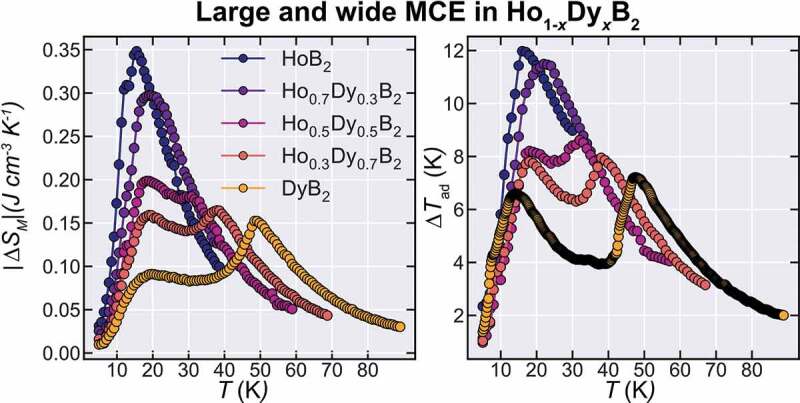

## 1. Introduction

Magnetic refrigeration is an emerging environmentally friendly technology for refrigeration applications, as it does not require to use of greenhouse gases and does not depend on conventional gas compression cycles [1–3] while having possible higher cycle efficiency [1,4]. It is based on the magnetocaloric effect (MCE), which consists of the adiabatic temperature change (Δ*T*_ad_) a magnetic material will undergo when a magnetic field is applied/removed adiabatically, but it can also be evaluated in terms of the magnetic entropy change (Δ*S*_M_) this magnetic material will undergo for the same field change, where Δ*S*_M_ usually peaks at the magnetic transition temperature (*T*_mag_).

Recently, our group has unveiled a giant magnetocaloric effect of |Δ*S*_M_^MAX^| = 0.35 J cm^−3^ K^−1^ (40.1 J kg^−1^ K^−1^) in the vicinity of a ferromagnetic transition at the Curie temperature (*T*_C_) of 15 K for a field change of μ_0_Δ*H* = 5 T in HoB_2_ [5]. Due to the closeness of its *T*_C_ to the liquefaction point of hydrogen (20.3 K), this material became an attractive candidate for use in low-temperature magnetic refrigeration applications focused on the liquefaction stage of hydrogen. Hydrogen is considered to be one of the most promising replacements for hydrocarbon fuels as a clean energy source [6,7] and in particular liquid hydrogen is widely needed in the space industry [8] and its liquid form is one the suitable way for transportation and storage [9]. In this context, the discovery of magnetic materials with a high MCE effect at low temperatures is imperative for the development of such refrigerators working at cryogenic temperatures. Since the magnetocaloric effect peaks at *T*_mag_, tuning the *T*_C_ of HoB_2_ to a higher temperature is of extreme interest to examine HoB_2_-based materials as possible candidates for refrigeration before the liquefaction stage, especially below temperatures of 77 K.

DyB_2_ orders ferromagnetically at *T*_C_ = 50 K [10,11] and exhibits a |Δ*S*_M_| of 0.16 J cm^−3^ K^−1^ (17.1 J kg^−1^ K^−1^) for μ_0_Δ*H* = 5 *T* [12]; 9 therefore a partial substitution of Ho by Dy is expected to shift *T*_C_ to higher values in the expense of a probable reduction of |Δ*S*_M_|. Also, since both materials exhibit two consecutive transitions, it is interesting to investigate the effect of alloying in the MCE properties of this system. In this work, we study the magnetocaloric properties of Ho_1-*x*_Dy*_x_*B_2_ alloys (*x* = 0, 0.3, 0.5, 0.7, 1.0) and compare with other well-known materials working at the same temperature span.

All the magnetocaloric properties of the samples are reported in volumetric units (J cm^−3^ K^−1^) as this is the adequate unit when comparing materials for application purposes as there is a volume limit when constructing real applications [13,14]. Therefore, herein all comparisons with other materials is done in this unit by converting it using the ideal density of each material when not provided.

## 2. Experimental section

### 2.1. Sample synthesis

Polycrystalline samples of Ho_1-*x*_Dy*_x_*B_2_ were prepared by an arc-melting process in a water-cooled copper hearth arc furnace under Ar atmosphere. Stoichiometric amounts of Ho (99.9% purity), Dy (99.9% purity), and B (99.5% purity) were weighted and then arc melted several times. During the synthesis trials, we found out that annealing under different conditions did not change the X-ray diffraction patterns of the obtained samples, therefore no annealing was carried out in this work.

### 2.2. Characterization

Powder X-ray diffraction (XRD) patterns of the arc-melted samples were investigated using a MiniFlex 600 (Rigaku, Japan) with Cu Kα radiation. The lattice parameters, the volume of the unit cell, and density were obtained by refining the XRD patterns using the FULLPROF [15] software.

### 2.3. Magnetization measurements

Magnetization measurements were carried out by a superconducting quantum interference device magnetometer contained in the Magnetic Property Measurement System XL (Quantum Design, US). Zero-field cooling (ZFC) and field cooling (FC) measurement at low fields were taken to evaluate the evolution of *T*_C_ as a function of Dy content. For the evaluation of |Δ*S*_M_| the magnetization measurements of the sample under various applied fields ranging from 0.01 to 5 T were performed in ZFC process.

## 3. Results and discussion

### 3.1. Crystal structure

**Figure 1.**
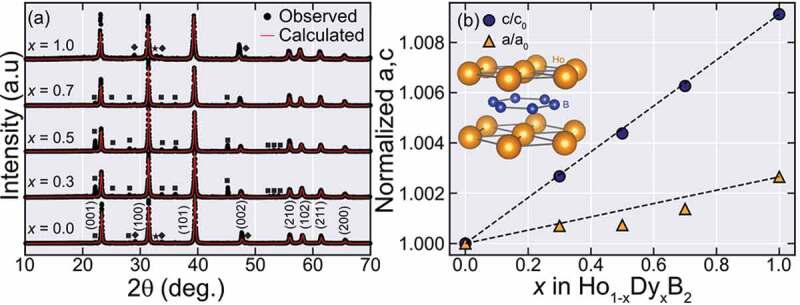
Powder XRD patterns and lattice constant evolution for Ho_1-*x*_Dy*_x_*B_2_ alloys. (a) XRD patterns of the obtained alloys. The red lines show the calculated patterns from Rietveld refinement for the REB_2_ main phase. The black square (♦) marks an REB_4_ impurity phase, while the black star (★) marks a RE impurity peak and the black diamond (♦) marks a RE_2_O_3_ impurity peak (RE = Ho, Dy). (b) The lattice parameters normalized by the value at *x* = 0, as a function of *x*. The black dashed line shows a guide based on Vegard’s law, given by $1-x+x\left(\frac{a_{1},c_{1}}{a_{0},c_{0}}\right)$ where (a,c)_0,1_ is the lattice constants at *x* = 0 or 1

Figure 1(a) shows the XRD patterns for the obtained arc melted samples. The main phase peaks can be indexed into a hexagonal *P*6/*mmm* AlB_2_ type crystal structure as shown by the red fitting curves. The remaining peaks are assigned as REB_4,_ unreacted RE or RE_2_O_3_ (RE = Ho, Dy) impurity peaks marked by a black square (∎), a black star (★), or a black diamond (♦) respectively. The obtained lattice parameters, the volume of the unit cell, and density are summarized in Table 1.

**Table 1.** Lattice parameters obtained from XRD patterns for Ho_1-*x*_Dy*_x_*B_2._

Nominal Dy (x)	a (Å)	c (Å)	V (Å^3^)	ρ (g/cm^3^)
0	3.283(3)	3.815(8)	35.62(4)	8.696
0.3	3.285(6)	3.827(1)	35.77(8)	8.626
0.5	3.285(7)	3.832(5)	35.82(9)	8.591
0.7	3.287(8)	3.839(7)	35.94(7)	8.540
1.0	3.292(0)	3.850(6)	36.14(0)	8.461

As shown in Table 1, Dy substitution in the Ho site seems to strongly affect the *c-*axis length while the *a*-axis length weakly changes, illustrated in Figure 1(b) where we plot the normalized lattice parameters (*a/a_0_* and *c/c_0_*) by the value of *x* = 0. Both *c/c_0_* and *a/a_0_* increase with *x*, roughly following the so-called Vegard’s law (marked by the dashed black line), but with different rates. The observed changes in the lattice constants in Ho_1-*x*_Dy*_x_*B_2_ suggest that the substitution of Ho by Dy in the REB_2_ main phase was successful, and these partially substituted samples can be in the form of a random alloy. We note that in the case of HoB_2-*x*_Si*_x_* solid solutions [10] where B site is partially substituted, it has been reported that the expansion rate of *a*-axis length and *c*-axis length are comparable to each other. This difference in the change of lattice constants between Ho_1-*x*_Dy*_x_*B_2_ and HoB_2-*x*_Si*_x_* implies that the *a*-axis and *c-*axis lengths in HoB_2_-based compounds might be closely related to the bonds along axes. Namely, *c*-axis length seems to be depending on Ho-B bonds and be sensitive to both rare-earth and B-site atoms, while the *a*-axis length might be more dependent on the B site atom.

### 3.2. Magnetic properties

**Figure 2.**
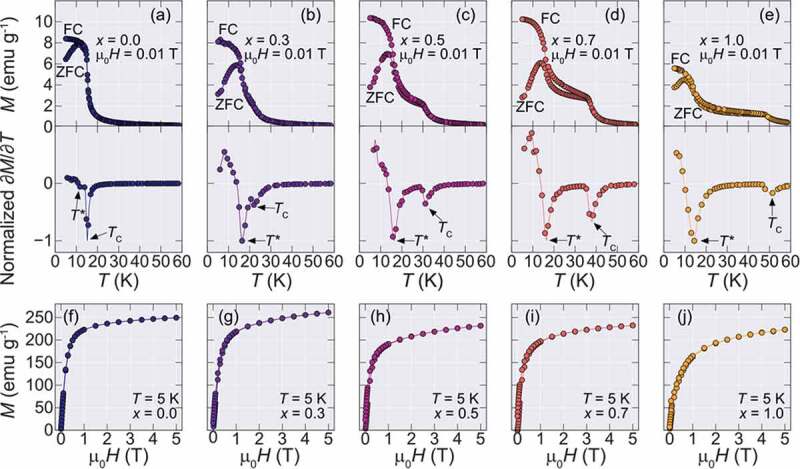
Isofield (*M-T)* ZFC-FC, Normalized temperature-dependent derivatives of the ZFC curves, and Isothermal (*M-H*) magnetization curves of Ho_1-x_Dy_x_B_2_ alloys. (a-e) ZFC and FC curves for all synthesized alloys for an applied field of µ_0_*H* = 0.01 T. The lower panels show the derivatives of the ZFC curves normalized by the minimum of the derivative value. The two magnetic transitions *T*_C_ and T*, are marked by the arrows. (f-g) Isothermal magnetization at *T* = 5 K

The ZFC-FC isofield magnetization (*M-T*) curves for an applied field of µ_o_*H* = 0.01 T and isothermal magnetization (*M-H*) curves measured at *T* = 5 K are shown in Figure 2(a-e, f-g) for each obtained sample, respectively. For the Dy containing samples, the divergence between the ZFC and FC *M-T* curves becomes more pronounced and a small magnetic hysteresis in the *M-H* curves is observed, including the end-material DyB_2_.

**Figure 3.**
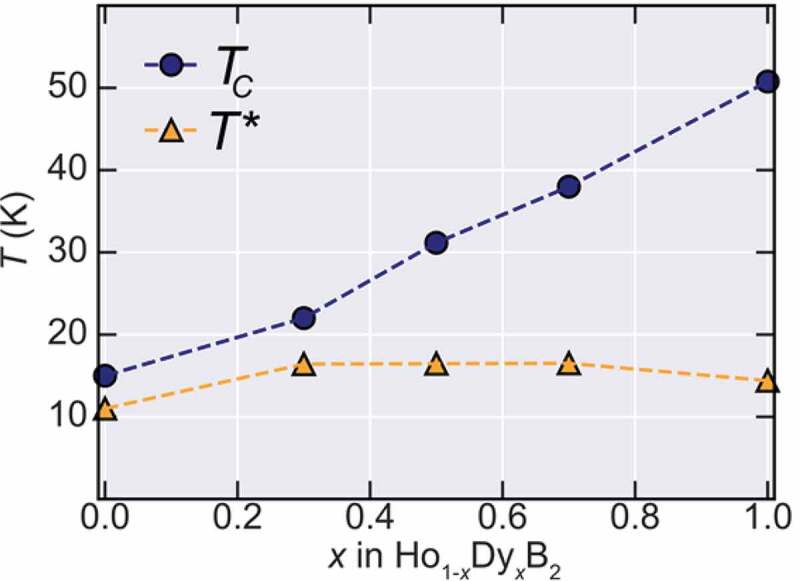
Phase diagram between ordering temperature and doping amount (*x*) for Ho_1-*x*_Dy*_x_*B_2_. The blue filled circles show the evolution of *T*_C_ while the orange triangles show T*. While *T*_C_ increases monotonically with x, T* remains almost constant

To evaluate the magnetic transition temperatures in this system, the temperature-dependent derivative of the ZFC curves was taken and are shown in the lower panels of Figure 2(a-e). The Curie temperatures that are defined by the peak position in ∂M/∂T curves, are marked by the *T*_C_ arrows, showing a systematic increase with Dy content. On the other hand, a second magnetic transition marked by T* that is observed at lower temperatures, which is also observed at HoB_2_ at T* = 11 K [5] and DyB_2_ at T* = 15 K [12], seems to be almost unchanged by partial substitution of Dy. The origin of T* was attributed to a possible spin-reorientation mechanism [12], however, the nature of this transition is still unknown and its investigation is outside the scope of this work. The Dy doping dependence of both transitions is summarized in Figure 3 showing the monotonic increase of *T*_C_ until 50 K, while T* remains almost constant.

### 3.3. Magnetocaloric properties

**Figure 4.**
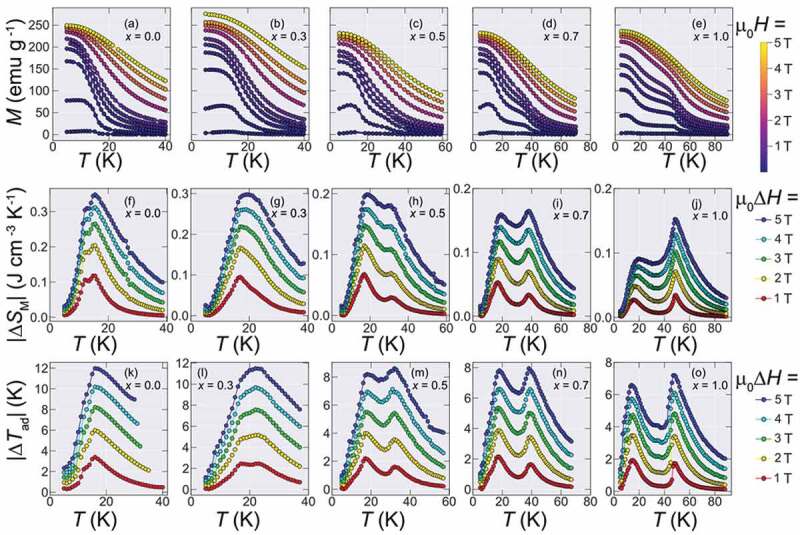
*M-T* curves at a vast range of applied fields and obtained magnetic entropy change for Ho_1-*x*_Dy*_x_*B_2_ alloys. (a-e) The obtained M-T curves measured by ZFC process from µ_0_*H* = 5 T to 0.01 T. (f-j) Magnetic entropy changes for Ho_1-*x*_Dy*_x_*B_2_ alloys for µ_0_Δ*H* ranging from 1 to 5 T obtained from the *M-T* curves of (a-e). (k)-(o) Estimated adiabatic temperature change for Ho_1-*x*_Dy*_x_*B_2_ alloys for µ_0_Δ*H* ranging from 1 to 5 T [21]. With the increase of Dy content, the maximum value of |Δ*S*_M_| decreases from 0.35 J cm^−3^ K^−1^ (*x* = 0) to 0.16 J cm^−3^ K^−1^ (*x* *=* 1.0)

For evaluating the magnetocaloric effect of the obtained samples, *M-T* curves in a wide range of applied magnetic fields were measured for all samples, shown in Figure 4(a-e), and |Δ*S*_M_| was calculated using the Maxwell relation:

$$\;\;\;\;\;\;\;\;\;\;\;\;\;\;\Delta S_{M}=\mu_{0}\int_{0}^{H}\;{\left(\frac{\partial M}{\partial T}\right)}_{H}dH\;\;\;\;\;\;\;\;\;\;\;\;\;\;\;\;\;\;\;\;\;\;\;\;\;\;\;\;\;\;\left(1\right)$$

The obtained |Δ*S*_M_| for fields up to 5 T is shown in Figure 4(f-j).

Due to the presence of the two transitions at T* and *T*_C_, two peaks appear at |Δ*S*_M_|. Therefore, here we will define and compare the maximum entropy change |Δ*S*_M_^MAX^| as |Δ*S*_M_(*T* = *T_C_*)|, since T* remains almost unchanged during the whole doping range and is always lower than 15 K while we are interested in the |Δ*S*_M_| peak shifted toward higher temperature by Dy doping. In this way, the obtained values of |Δ*S*_M_^MAX^| for µ_0_Δ*H* = 5 T were 0.35, 0.3, 0.18, 0.16 and 0.15 J cm^−3^ K^−1^ for *x* = 0, 0.3, 0.5, 0.7 and 1.0, respectively.

**Figure 5.**
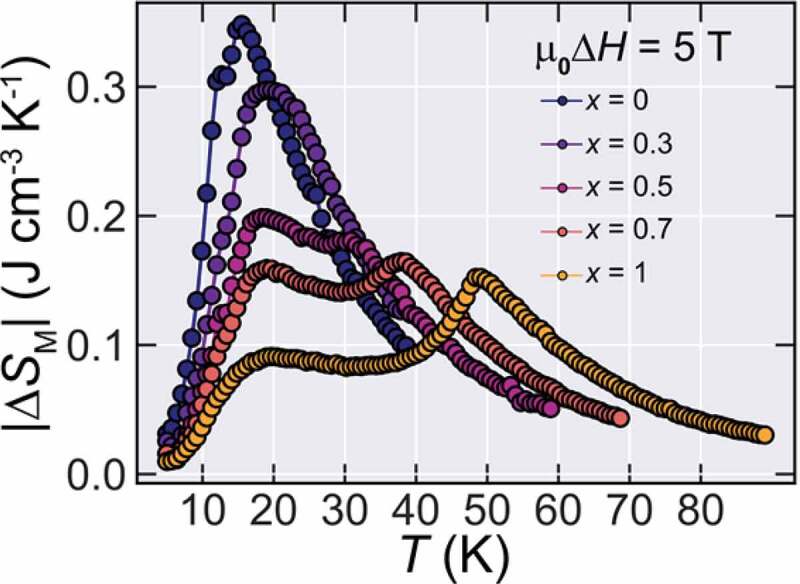
|Δ*S*_M_| at µ_0_Δ*H* = 5 T for Ho_1-*x*_Dy*_x_*B_2_ alloys

In addition to the change in the magnitude of |Δ*S*_M_^MAX^|, an interesting characteristic appears in the |Δ*S*_M_| curves of Ho_1-*x*_Dy*_x_*B_2_. That is, since there are multiple transitions in this series of alloys, even though there is a net loss at |Δ*S*_M_^MAX^|, the entropy change curve shows an increase of δ*T*_FWHM_, defined as the region in the entropy curve where |Δ*S*_M_| ≥ |Δ*S*_M_^MAX^|/2, leading to a gain in maximum entropy change for higher temperature spans. Such a widening of the |Δ*S*_M_| curves due to multiple transitions has been commonly observed in materials that show more than one magnetic transition [16–18] and it tends to lead to a high figure of merits. The |Δ*S*_M_| for all samples for a field change of μ_0_Δ*H* = 5 T is shown in Figure 5.

Another important property in the magnetocaloric performance of materials is the adiabatic temperature change Δ*T*_ad_. Here, we estimated the *S*(*T, H*) (shown in Figure S1.) curves by first calculating the zero-field entropy from specific heat data (not shown here) and then we obtain the field-dependent entropy by subtracting the values of |Δ*S*_M_| to the zero-field entropy, in the same manner as Refs. [5,19]. For the *x* = 0, we used the previously reported data of Ref [5]., and for the *x* = 1 sample, we used the previously reported zero-field specific heat data of Ref [20]. Then, the Δ*T*_ad_s is estimated by taking the horizontal difference between the entropy curves under zero field and final field [21] and the results are shown in Figure 4(k-o). Interestingly, the two-peak structure of |Δ*S*_M_| is also reflected in Δ*T*_ad_ and thus these alloys tend to show high Δ*T*_ad_ in relatively wide temperature range, that is an important characteristic for practical applications [22]. Here we estimated Δ*T*_ad_^MAX^ in the same way as |Δ*S*_M_^MAX^|, that is, Δ*T*_ad_^MAX^ = Δ*T*_ad_ (*T* = *T*_C_). The obtained Δ*T*_ad_^MAX^ is 12 K, 11.5 K, 8.6 K, 8.0 K and 7.2 K for *x* = 0, 0.3, 0.5, 0.7 and 1.0, respectively for µ_0_Δ*H* = 5 T.

Let us compare the magnetocaloric properties in Ho_1-x_Dy_x_B_2_ with those of representative materials that often show prominent magnetocaloric effect with transition temperatures ranging up to 77 K, based on Figure S5 of Ref [5]. Here we consider Δ*S*_M_, Δ*T*_ad_, and Temperature averaged Entropy Curve (TEC) as a practical figure of merit proposed earlier [23]. The last value is defined as:

$$TEC\left(\Delta T_{lift}\right)=\frac{1}{\Delta T_{lift}}\underset{T_{mid}}{\max}\int_{T_{mid}-\frac{\Delta T_{lift}}{2}}^{T_{mid}+\frac{\Delta T_{lift}}{2}}\Delta S_{M}dT$$

Where *T*_mid_ is chosen to maximize the value of *TEC* in a given working temperature range of a material (Δ*T*_lift_) (Conventional figure of merits such as refrigerant capacity and relative cooling power are also tabulated in supplementary information).

**Figure 6.**
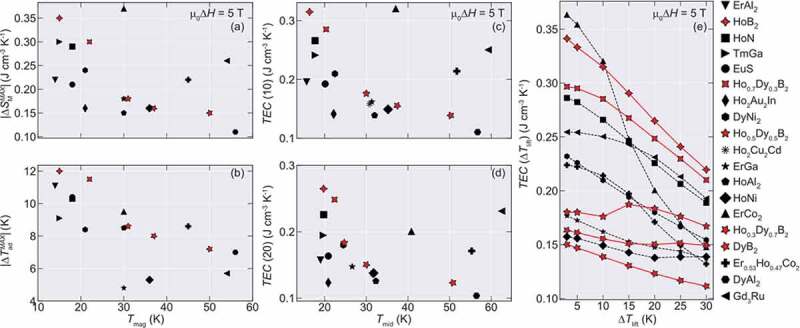
Maximum entropy and adiabatic temperature change and TEC values for Ho_1-*x*_Dy*_x_*B_2_ alloys and diverse compounds for µ_0_Δ*H* = 5 T. (a) |Δ*S*_M_^MAX^| and (b) |Δ*T*_ad_^MAX^| as a function of the magnetic ordering temperature *T*_mag_. (c) Values of TEC for Δ*T*_lift_ = 10 K and (d) for Δ*T*_lift_ = 20 K. (e) TEC as a function of Δ*T*_lift_ for few representative materials with high TEC 10 values. The normal and dashed lines guide for the eyes. The TEC values were obtained by using the reported entropy curves within each material reference

For this purpose, the values of entropy change of the materials for comparison are converted into volumetric units by using the density contained in the AtomWork [24] database, unless otherwise provided by the authors. Also, the values of *TEC* (10) and *TEC* (20) are estimated from the reported entropy curves within the contained references when not reported by the authors. We show the obtained values for |Δ*S*_M_^MAX^|, |Δ*T*_ad_^MAX^_|_, *TEC* (10), and *TEC* (20) in Figure 6(a-d). Also, the Δ*T*_lift_-dependence of *TEC* in selected materials is shown in Figure 6(e).

In the temperature range of 15–20 K, HoB_2_ and Ho_0.7_Dy_0.3_B_2_ show superior |Δ*S*_M_^MAX^| and Δ*T*_ad_^MAX^ for µ_0_Δ*H* = 5 T, when compared to compounds with similar *T*_mag_ such as ErAl_2_ [25], TmGa [26], EuS [27], HoN [28,29], DyNi_2_ [25] and Ho_2_Au_2_In [30]. For the materials with transition temperatures around 30 K, even though Ho_0.5_Dy_0.5_B_2_ shows similar |Δ*S*_M_^MAX^| to Ho_2_Cu_2_Cd [18] and ErGa [31], it has a comparable |Δ*T*_ad_^MAX^| to HoAl_2_ [32] and ErCo_2_ [33]. Similarly, Ho_0.3_Dy_0.7_B_2_ has almost the same |Δ*S*_M_^MAX^| as HoNi [34,35], however with a much larger |Δ*T*_ad_^MAX^|. In the case of DyB_2_, even though it shows almost half of |Δ*S*_M_| compared to Gd_3_Ru [36], its |Δ*T*_ad_^MAX^| is higher and comparable to DyAl_2_ [37], although both of them are lower than Er_0.53_Ho_0.47_Co_2_ [38,39]. Furthermore, the TEC in Ho_1-*x*_Dy*_x_*B_2_ alloys tends to be relatively robust for a higher value of Δ*T*_lift_, due to the multiple transition nature in these materials. This suggests that Ho_1-*x*_Dy*_x_*B_2_ alloys could support a wide temperature span while keeping the figure of merit and indicate that Ho_1-*x*_Dy*_x_*B_2_ alloys might be an option for use in magnetic refrigeration ranging from 10 to 60 K as similar materials with *T*_mag_ within this range.

## 4. Conclusions

In this work, we have systematically evaluated the effects of Dy substitution on the giant magnetocaloric effect of HoB_2_. Even if there is a net loss in the peak value of the |Δ*S*_M_|, the observed two-peak structure in both the magnetic entropy and adiabatic temperature change might indicate these materials could possibly sustain a large working temperature range based on the figure of merit analysis. Therefore, these alloys could be an option to work as magnetic refrigerants in the temperature range from 10 to 60 K.
